# Evaluating Barriers and Facilitators to the Uptake of mHealth Apps in Cancer Care Using the Consolidated Framework for Implementation Research: Scoping Literature Review

**DOI:** 10.2196/42092

**Published:** 2023-03-30

**Authors:** Vittoria Ardito, Georgi Golubev, Oriana Ciani, Rosanna Tarricone

**Affiliations:** 1 Centre for Research on Health and Social Care Management (CERGAS) Government, Health and Not for Profit Division SDA Bocconi School of Management Milan Italy; 2 Department of Social and Political Science Bocconi University Milan Italy

**Keywords:** mobile health, mHealth, smartphones, mobile, oncology, cancer, implementation science, consolidated framework for implementation research, CFIR, mobile phones

## Abstract

**Background:**

Mobile health (mHealth) solutions have proven to be effective in a wide range of patient outcomes and have proliferated over time. However, a persistent challenge of digital health technologies, including mHealth, is that they are characterized by early dropouts in clinical practice and struggle to be used outside experimental settings or on larger scales.

**Objective:**

This study aimed to explore barriers and enablers to the uptake of mHealth solutions used by patients with cancer undergoing treatment, using a theory-guided implementation science model, that is, the Consolidated Framework for Implementation Research (CFIR).

**Methods:**

A scoping literature review was conducted using PubMed (MEDLINE), Web of Science, and ScienceDirect databases in March 2022. We selected studies that analyzed the development, evaluation, and implementation of mHealth solutions for patients with cancer that were used in addition to the standard of care. Only empirical designs (eg, randomized controlled trials, observational studies, and qualitative studies) were considered. First, information on the study characteristics, patient population, app functionalities, and study outcomes was extracted. Then, the CFIR model was used as a practical tool to guide data collection and interpretation of evidence on mHealth uptake.

**Results:**

Overall, 91 papers were included in the data synthesis. The selected records were mostly randomized controlled trials (26/91, 29%) and single-arm, noncomparative studies (52/91, 57%). Most of the apps (42/73, 58%) were designed for both patients and clinicians and could be used to support any type of cancer (29/73, 40%) and a range of oncological treatments. Following the CFIR scheme (*intervention*, *outer setting*, *inner setting*, *individuals*, *process*), multistakeholder co-design, codevelopment, and testing of mHealth interventions were identified as key enablers for later uptake. A variety of external drivers emerged, although the most relevant outer incentive fostering mHealth use was addressing patient needs. Among organizational factors likely to influence technology uptake, interoperability was the most prominent, whereas other providers’ dimensions such as managerial attitudes or organizational culture were not systematically discussed. Technology-related impediments that could hamper the use of mHealth at the individual level were considered least often.

**Conclusions:**

The hype surrounding mHealth in cancer care is hindered by several factors that can affect its use in real world and nonexperimental settings. Compared with the growing evidence on mHealth efficacy, knowledge to inform the uptake of mHealth solutions in clinical cancer care is still scarce. Although some of our findings are supported by previous implementation research, our analysis elaborates on the distinguishing features of mHealth apps and provides an integrated perspective on the factors that should be accounted for implementation efforts. Future syntheses should liaise these dimensions with strategies observed in successful implementation initiatives.

## Introduction

### Background

Mobile health (mHealth) apps, defined by the World Health Organization as “medical and public health practice supported by mobile devices, such as mobile phones, patient monitoring devices, personal digital assistants, and other wireless devices” [1], have become increasingly relevant in the health arena since the introduction of smartphones in 2007 [2]. With >6 billion smartphone users, indicating a penetration rate of >78% by the end of year 2020 [3], the number of mHealth apps has been increasing exponentially over time, leading to >351,000 mHealth apps available in the market in 2021 [4]. The COVID-19 outbreak accelerated this pattern, and mHealth provided a valid opportunity to deliver care remotely [5-7].

In oncology, mHealth apps have shown to provide benefits to patients throughout the care pathway [8-10]. Cancer treatments are complex, and mHealth apps can help patients manage their therapy more effectively and efficiently [11] by enabling better collection of patient data, remote monitoring by clinicians, patient education, and user-friendly communication tools [12]. In addition, apps have been shown to increase medication adherence, leading to reduced adverse events and increased quality of life [13,14]. This is particularly helpful for patients undergoing oral anticancer treatments, often performed in outpatient settings, whose success relies heavily on patients’ treatment compliance [15]. Overall, mHealth apps have the potential to increase patient empowerment by enhancing self-efficacy and improving patient-physician interaction [16].

Not only do individual patients benefit from using mHealth solutions, but also the broader health care system. There is a growing interest in the uptake of mHealth solutions in clinical practice because they have the potential to offer more accessible and cost-effective health care solutions [17]. Compared with conventional in-person therapies, mHealth can reduce health care costs while maintaining the same treatment quality by allowing the patient to attend follow-up appointments remotely [18,19]. By reducing commuting to and from the hospital, mHealth also holds great promise in mitigating the environmental impact of health care delivery [20], as commented by a recent study that appraised the potential environmental impact as a distinguished outcome domain of mobile medical apps [21].

The potential of mHealth is also reflected at the policy level, with an increasing number of countries gradually adopting regulatory frameworks [22]. For instance, the mHealthBelgium framework allows systemized recognition of mHealth apps as a medical device [23] using 3 validation levels depending on the safety level and socioeconomic value. Apps labeled with a level 3 status can be refunded by the National Institute for Health and Disability Insurance [24]. Similarly, in Germany, patients can apply for reimbursement of an mHealth app as a part of their statutory health insurance scheme if it is certified under Digital Health Applications (DiGA) regulation [25]. As of May 2022, the only DiGa-certified mHealth app for cancer care is CANKADO PRO-React Onco, which provides digital support to patients undergoing cancer treatment by facilitating communication with physicians and promoting patient education and empowerment [26]. In France, although some apps already receive reimbursement (eg, MOOVCARE POUMON for lung cancer telemonitoring) [27,28], the government is working on an assessment framework similar to that of the German DiGa [29]. In England, the National Institute for Health and Care Excellence developed an evidence-based standards framework for digital health technologies (DHTs), which is intended to be used by both technology developers and decision makers to inform the evidence development plans of the technology developers and commissioning of DHTs from the decision makers [30]. In this context, the European Union has recently launched a task force with the mission of harmonizing the evaluation of digital medical devices [31].

Increased interest in mHealth in cancer care has been observed in the fast-growing number of scientific publications in the past few years. However, most studies have investigated the impact of mHealth apps on patient outcomes. For instance, recent literature reviews have assessed the effect of mHealth apps on pain management in patients with cancer [32-35]. Other studies have investigated the impact of mHealth apps on patients’ quality of life, satisfaction with care, and user acceptance. However, there is limited evidence on the impact of the uptake and use of mHealth apps within the clinical setting. DHTs, including mHealth, are challenged by the phenomenon of early dropouts and abandonment [36]. To date, the implementation of mHealth apps has been analyzed less extensively. Does mHealth guarantee time and monetary savings for both patients and health care providers? Are mHealth apps used beyond the controlled study settings? In this context, implementation science is defined as “applied research that aims to develop the critical evidence base that informs the effective, sustained, and embedded adoption of interventions by health systems and communities” [37]. Through an extensive set of validated frameworks, tools, and strategies, this study investigates barriers and enablers to implementation that, respectively halt or facilitate the actual uptake of clinically proven interventions.

### Objectives

Therefore, this study aimed to investigate the determinants of mHealth uptake using a theory-guided framework from implementation science, the Consolidated Framework for Implementation Research (CFIR). The CFIR was intended as a practical tool to map and interpret empirical evidence regarding factors (ie, barriers and facilitators) that could affect the implementation of mHealth in cancer care.

## Methods

### Study Design

This review follows the updated methodological guidance for scoping reviews [38] and the PRISMA-ScR (Preferred Reporting Items for Systematic Reviews and Meta-Analyses extension for Scoping Reviews) guidelines [39]. Scoping reviews aim to identify the main concepts, theories, sources, and knowledge gaps regarding a given topic of interest. The study protocol has not been registered. The 22-item PRISMA-ScR checklist for scoping reviews is provided in Multimedia Appendix 1.

### Search Strategy

Web of Science, PubMed (MEDLINE) and ScienceDirect were consulted. The search was extended to the papers published from January 2017 to March 2022. A 5-year timeframe was deemed appropriate considering the sharp increase in the number of studies on the topic and the rapid obsolescence of previous studies. Additional relevant studies were identified by screening the bibliographies of other published reviews (snowballing).

The search strategy was defined jointly by the research team and ultimately built around 2 broad content areas, cancer and mHealth. The exact keyword string used was as follows: (cancer OR tumor OR tumour OR oncolog*) AND (mHealth OR “mobile health” OR phone OR smartphone OR app). The search was restricted to titles and abstracts in PubMed, and to titles, abstracts, and keywords in Web of Science and ScienceDirect.

RefWorks [40] was used to retrieve relevant information from articles that were later exported in Microsoft Excel form for articles screening and data extraction. All papers selected for full-text reading were handled by the bibliographic reference manager, Zotero [41].

### Eligibility Criteria

Only empirical study designs describing the development, evaluation (including testing), and implementation of an mHealth intervention were included. Other study types, including literature reviews, meta-analyses, conference abstracts, and clinical guidelines, were excluded. Studies were included if they focused on mHealth apps used as support for ongoing cancer therapies or management of related adverse events. Typical app functionalities included, but not limited to, enhancing patient self-monitoring, self-efficacy, or education, as well as fostering patient-clinician communication. Conversely, studies assessing mHealth apps used in other phases of the care pathway (eg, screening, diagnosis, and palliative care) were excluded. mHealth apps exclusively delivering noncore ancillary services for patients with cancer (eg, mental health, physical activity, and smoking cessation) were also out of scope. As for the target mHealth users, only adult patients undergoing cancer treatment were considered, whereas studies on cancer survivors, pediatric populations, or other targets with risky conditions or behaviors (eg, comorbidities) were excluded. Finally, studies not published in English were excluded. A detailed illustration of the inclusion and exclusion criteria is provided in Textboxes 1 and 2.

**Inclusion criteria for paper selection.**

Study design

- Empirical studies (eg, randomized controlled trials, observational studies, pre-post studies, and qualitative designs)

App functionality

- Mobile health apps facilitating core cancer treatment delivery (eg, symptom-monitoring, tele-visit, and communication with health care professionals)

Moment of care

- Mobile health apps used as a support to ongoing cancer therapies or related adverse events

Target population

- Adult patients undergoing cancer treatment

Publication language

- English

Publication year

- From 2017 (included)

**Exclusion criteria for paper selection.**

Study design

- Literature review, meta-analysis, conference abstract, and clinical guideline

App functionality

- Mobile health apps exclusively delivering noncore, ancillary services for cancer patients (eg, exercise programs)

Moment of care

- Other phases of the care pathway (eg, screening and prevention, diagnosis, and palliative care)

Target population

- Cancer survivors, pediatric populations, or other targets with risky conditions (eg, multimorbidities) or behaviors (eg, smokers)

Publication language

- Any other language except English

Publication year

- Before 2017

### Study Selection

After double-checking a sample with a second reviewer (VA), the researcher GG screened all retrieved articles based on title and abstract, whereas full-text reading was performed by GG and VA. Disagreements regarding the inclusion of a given article were resolved by a third researcher (RT). All researchers agreed on the final selection of the studies selected for data synthesis. Owing to the variety of included studies in terms of design, objectives, and sources of evidence, no assessment of the risk of bias or methodological quality was undertaken.

### Data Extraction and Analysis

Data extraction was performed in a Microsoft Excel grid. The extracted data included a general overview of the studies (eg, publication country, study objective, design, and duration), information on study participants (eg, number of participants, age, cancer type and stage, and cancer treatment), information on mHealth apps (eg, use time, app name, and main functionalities), study outcomes, and related metrics. The taxonomy by Dodd et al [42] that classifies the outcomes in medical research, was used to cluster the apps in the selected studies based on the investigated outcomes. In addition, CFIR was used to guide data collection and analysis of enablers and barriers to mHealth implementation, as well as strategies to overcome them. CFIR encompasses 5 domains and 39 constructs associated with effective implementation [43]. CFIR acts as a practical guide for systematically assessing potential barriers and facilitators when implementing innovation. CFIR integrates perspectives from different stakeholders and settings without inferring assumptions or drawing conclusions about the mechanisms of implementation, which is well suited to the heterogeneous literature to be synthesized [44]. A comprehensive explanation of the CFIR variables is provided in Multimedia Appendix 2.

The results were summarized using mainly a narrative synthesis and organized into 2 major sections. First, an overview of the selected studies and underlying app functionalities was provided, including key statistics (eg, count and proportions) and summary characteristics when relevant. Evidence on barriers and enablers specific to mHealth implementation was then analyzed following the CFIR framework. We did not expect to find evidence on every CFIR subdomain in each selected study; therefore, data analysis was conceived as a synthesis of subsets of relevant, available observations.

## Results

### Review Profile

**Figure 1:**
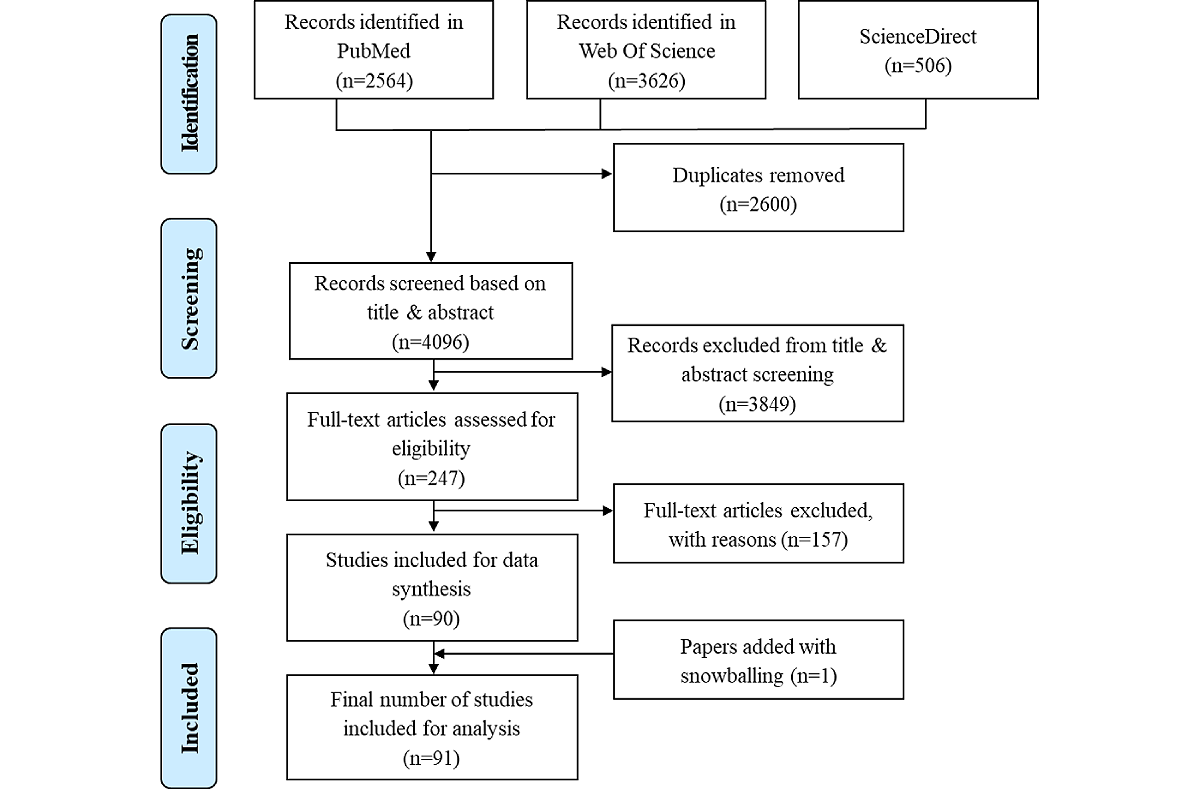
PRISMA (Preferred Reporting Item for Systematic Reviews and Meta-Analyses) flowchart.

A total of 6190 papers were identified through the search (2564 records from PubMed, 3626 from Web of Science, and 506 from ScienceDirect). After duplicate removal, 3915 records remained for screening based on the title and abstract. A final number of 91 studies were included for analysis. Figure 1 describes the PRISMA flowchart [36,37].

### Overview of Selected Studies

Of the 91 studies, 78 (86%) [8,9,12,16,45-118] were research articles, whereas 13 (14%) [119-131] were study protocols. From 2017 to 2022, the number of published articles increased steadily over time. Almost half of the studies (43/91, 47%) were published in Europe, with Sweden (9/91, 10%), the United Kingdom (7/91, 8%), and Germany (6/91, 7%) having the highest number of publications. Outside Europe, relevant studies on mHealth in oncology were conducted in the United States (18/91, 20%), China (9/91, 10%), and South Korea (6/91, 7%).

In terms of study designs, randomized controlled trials (RCTs), including secondary analyses of RCT data, were the most common (26/91, 29%), followed by mixed-methods studies (24/91, 26%), qualitative design studies (12/91, 13%), pilot studies (11/91, 12%), other non-RCTs (7/91, 8%), pre-post studies (3/91, 3%), quasi-experimental studies (3/91, 3%), and other study designs (5/91, 6%). The majority (52/91, 57%) were single-arm studies, whereas 43% (39/91) of the studies were comparative, with 2 or multiple arms. Most of the included studies had a prospective design (84/91, 92%), 3 were retrospective, and others were combined retrospective and prospective branches (4/91, 4%).

Owing to their heterogeneous nature, the selected articles had different study durations, ranging from 2 weeks for small-scale trials to up to 2 years for larger-scale RCTs. The median sample size of the study participants was 51, ranging from a minimum of 5 to a maximum of 4475 patients.

Multimedia Appendices 3 and 4 provide an overview of the descriptive statistics and detailed study characteristics in a tabular format.

The 91 studies included for analysis describe 73 mHealth apps, of which 29 (40%) were designed for supporting any cancer types [9,12,47,48,50,54,56-58,65-69,71,73,74,76,81-83,85-88,​^90^,^91^,^93^,^105^-^109^,^111^-^113^,^116^,^117^,^121^,^122^,^125^,^128^], followed by 17 (23%) on breast cancer [49,64,70,72,77,​^78^,^80^,^95^-^98^,^103^,^114^,^115^,^118^,^124^,^126^,^129^-^131^], 5 (7%) [52,53,63,99,101,102] on gastric and colon cancer types, 3 (4%) [75,79,89,119] on lung cancer, 3 (4%) on thyroid cancer [84,100,123] type, and 2 (3%) on hematological cancer types [9,55,56,94,121]. The remaining apps (15/73, 21%) [8,16,45,46,51,59-62,92,104,110,118,120,127] covered other types of cancer, such as pancreatic, bone marrow, prostate, brain, and gynecological cancers.

Many apps did not support a specific cancer treatment (23/73, 32%) [46,51,53,62,71-74,83,84,86,88,93,95,98,99,104,105,108,​^112^,^117^,^118^,^121^,^124^,^126^]. The most frequent treatment specifications were chemotherapy (15/73, 21%) [47,50,55,63,64,70,78,85,87,101,110,114-116,119,127,129,131], oral anticancer treatments (13/73, 18%) [9,48,52,​^56^-^58^,^65^,^76^,^80^,^82^,^90^,^91^,^94^,^111^,^122^,^128^], radiotherapy (3/73, 4%) [54,92,120], and others (8/73, 11%) [12,16,45,59,60,66-69,75,81,89,102,106,107,113,125], which included several treatment types, such as a combination of chemotherapy and radiotherapy. Nonpharmacological treatments include surgery (8/73, 11%) [8,49,61,79,96,109,123,130] and transplantation (3/73, 4%) [77,97,100,103].

mHealth users can be patients, clinicians, a broader pool of health care professionals (HCPs), or different combinations of users. Most commonly, apps are designed for both patients and clinicians (42/73, 58%) [12,16,48-55,58,59,61,63,66,​^67^,^69^,^75^,^77^,^84^-^86^,^89^,^91^-^94^,^96^,^98^,^100^,^101^,^104^-^106^,^108^,^110^,​^112^-^116^,^119^,^120^,^122^-^125^,^127^-^129^,^131^], who typically access different interfaces and functionalities (eg, self-reporting function for patients, web-based dashboards with overview of patient activity for the clinicians). Only 32% (23/73) apps [8,47,60,64,65,68,70-73,76,78-83,87,88,95,97,99,102,103,107,111,​^121^,^126^,^130^] were designed for exclusive patient use. This is the case for certain medication adherence apps that focus mainly on providing reminders to patients [65,76,79,80,82,99,111]. The remaining apps (8/73, 11%) [9,45,46,56,​^57^,^62^,^74^,^90^,^109^,^118^] had diverse combinations of end users with patients, clinicians, caregivers, and pharmacologists. The app functionalities are listed in Table 1.

**Table 1:** Summary of app functionalities (n=73).

Characteristics of mHealth^a^ apps		n (%)
**App cancer targets**		
	Any cancer (ie, generic)	29 (40)
	Breast	17 (23)
	Gastric and colon	5 (7)
	Lung	3 (4)
	Thyroid	3 (4)
	Hematological	2 (3)
	Other forms of cancer	15 (21)
**Cancer treatment supported**		
	Not specified	23 (32)
	Chemotherapy	15 (21)
	Oral treatment	13 (18)
	Surgery	8 (11)
	Radiotherapy	3 (4)
	Transplantation	3 (4)
	Other	8 (11)
**Intended app users**		
	Patients and clinicians	42 (58)
	Patients only	23 (32)
	Patients, clinicians, and caregivers	3 (4)
	Patients and caregivers	2 (3)
	Other combinations	3 (4)

^a^mHealth: mobile health.

The selected studies assessed mHealth impact using a wide range of outcome metrics analyzed using the taxonomy by Dodd et al [42]. Outcomes most recurrently fall under the *Life impact* area, with 73 outcomes in the *Delivery of Care* outcome domain [8,9,12,16,45,46,48-55,57-63,65,67,70,71,73-87,90-92,​^94^-^104^,^107^-^116^,^119^-^122^,^128^-^131^], 37 in *Global quality of life* [8,57,60,62,64,65,68,72,75,78,84,86,88-91,93,97,101,103,​^105^,^106^,^112^,^114^,^115^,^119^-^121^,^123^-^131^], 16 in *Emotional functioning and well-being* [66,70,77,83,89,90,93,106,​^115^,^119^,^125^-^128^,^130^,^131^], 8 in *Physical functioning* [75-77,89,101,102,105,130], and 7 in *Social functioning* [66,69,84,115,125,126,131]. Within this core area, recurring metrics were the acceptability, usability, and feasibility of mHealth apps, which could be assessed either using validated questionnaires, or qualitatively, through study-specific questionnaires or interviews. Specifically, feasibility was assessed in 41% (37/91) studies, usability in 40% (36/91) studies, and acceptability in 35% (32/91) studies.

As for the *Physiological or clinical* area, 12 outcomes are *General outcomes* [65,70,76,77,89,96,103,115,125,127,130,131] and 4 relate to *Neoplasms: benign, malignant, and unspecified* [4,77,101,112]. As for the *Resource use* area, outcomes fall under *Hospital* (n=10) [49,59,64,65,79,89,97,120,124,125], *Societal burden* (n=7) [65,77,115,119,127,128,131], and *Economic* (n=1) [126] domains. *Adverse events* related outcomes were recorded 9 times [49,53,56,57,60,78,93,108,112] and *Mortality or survival* [97] once. The outcome core areas and domains are summarized in Table 2.

**Table 2:** Outcomes according to the taxonomy by Dodd et al [42].

Core area *and o*utcome domain			Count		Examples
**Mortality or survival**					
	1. Mortality or survival	1		Overall survival	
**Physiological or clinical**					
	9. General outcomes	12		MDASI^a^	
	16. Outcomes relating to neoplasms: benign, malignant and unspecified	4		LARS^b^	
**Life impact**					
	25. Physical functioning	8		KPS^c^	
	26. Social functioning	7		PAM-13^d^	
	28. Emotional functioning and well-being	16		HADS^e^	
	30. Global quality of life	37		EORTC QLQ-C30^f^	
	32. Delivery of care	73		SUS^g^	
**Resource use**					
	34. Economic	1		Health resource use (cost)	
	35. Hospital	10		Reduction in unexpected visits to ED^h^	
	37. Societal burden	7		MSPSS^i^	
**Adverse events**					
	38. Adverse events and effects	9		CTCAE^j^	

^a^MDASI: MD Anderson Symptom Inventory.

^b^LARS: low anterior resection syndrome score.

^c^KPS: Karnofsky Performance Status.

^d^PAM-13: Patient Activation Measure–13.

^e^HADS: Hospital Anxiety and Depression Scale.

^f^EORTC QLQ-C30: European Organization for the Research and Treatment of Cancer Quality of Life Questionnaire.

^g^SUS: System Usability Scale.

^h^ED: emergency department.

^i^MSPSS: Multidimensional Scale of Perceived Social Support.

^j^CTCAE: Common Terminology Criteria for Adverse Events.

### Determinants of mHealth Uptake

#### Intervention Characteristics

App characteristics are important predictors of intervention implementation in later stages. Regarding the *intervention source*, the literature reported that participating in the development phase increased the likelihood of later embracing the technology. Most analyzed apps have been developed collaboratively [8,53,55-58,62,72,73,75,77,87,101,103,104,107,​^109^,^114^,^116^-^118^,^122^,^127^,^131^], often including HCPs, potential patients, and external technology partners responsible for actual software development [56,66,71,78,89,97,105,125,127,129,130]. For instance, the development of eOncoSalud was carefully planned during a series of 7 nominal consensus meetings involving a wide range of stakeholders [56]. Similarly, Konsghaug et al [80] followed an iterative and stepwise development approach, with the interactions of partners from diverse disciplines. Others followed participatory design techniques to foster stakeholder’s acceptance of the mHealth intervention, thereby increasing the likelihood of successful app implementation [73,74,90,91,95,99,104,117,118]. Perceived ease-of-use has emerged as a decisive factor for app uptake [74], and involving many actors in the development could also contribute to user-friendly interfaces (*design quality and packaging*). Satisfaction with the app design was gauged using satisfaction and usability questionnaires. Subsequent software releases and updates in app versions [64] are among the most perceived *complexities* of smartphone apps. As patient data are extremely sensitive, mHealth apps have specific data protection requirements. For instance, Giannoula et al [123] discussed data privacy and integrity (eg, cryptographed clouds, app authentication verification, and standards to transfer clinical and administrative data among software apps) and commented on the need to address data confidentiality issues from the early development phase [123]. The experimental nature of many of the study designs included in the analyses signaled the willingness to follow rigorous scientific approaches. Moreover, most studies adopted small-scale pilots to test the intervention before the roll-out (*trialability*) [66,68,80,90,91,97,102,104,119]. Nevertheless, the vast majority of included studies were noncomparative, thus hindering the possibility of assessing their *relative advantage* compared with other solutions. Being often developed for the purpose of the study, most apps were fit for the study context (*adaptability*), although incompatibility with IT systems was often mentioned as a hindering factor. Finally, practically no study has reported on the intervention development *costs* or on the economic impact of app use on the organization.

#### Outer Setting

The surge in the use of mHealth has attained new social needs and external policy pressures. Nearly every study stems from well-identified *patient needs and resources*, which are mostly related to a general improvement of the therapeutic pathway by means of better cancer-related symptom management [12,51,55,62,66,71,76,83,87,100,125,128], pain reduction [45,68,105,112,125], enhanced treatment adherence [48,58,65,82,91,94,111], and improved quality of life [51,99,112,124]. Another drive for mHealth uptake highlighted in the analysis was the scarcity of resources from national health systems, which pushed health care providers and policy makers to seek alternative solutions to conventional care. For instance, Zhu et al [131] reported insufficient financial commitment to health care from the government, which emerged in shortages of oncologists and the unviability of traditional face-to-face consultations. Considering recent government cost-cutting reforms, mobile-based, low-cost technologies are said to be crucial to lessening health care spending [119].

To address these newly developed needs or emerging social pressures, *external policies and incentives* have been issued to directly or indirectly foster mHealth deployment while regulating its diffusion. Broadly speaking, recent policy changes appeared to be oriented toward shaping patient care with more patient-centric service designs and posed greater attention to quality of life as opposed to only treating illnesses [74]. Examples of direct provisions can be observed in the newly issued guidelines on the facilitation of innovation diffusion by the United States Oncology Nursing Society [54], which advocates for a more individualized approach to cancer care or the need to comply with the US Health Insurance Portability and Accountability Act requirements for mHealth [52,58,87,96,110]. Provisions that strive to enhance patient-clinician communication, such as the Swedish law on patient empowerment in health care management [66] that encourages patients to participate in decision-making and to receive better knowledge about the treatment, or recommendations aimed at supporting patient self-management, such as the National Institute of Health guidelines on integrating behavioral pain interventions into cancer treatment [77], also emerged as facilitators of mHealth.

Finally, *cosmopolitanism* and *peer pressure*, namely competitive pressures to adopt an intervention because other peers are already using it, can further push the implementation process. These dimensions were not observed, as most studies only described isolated case studies and were carried out at single research centers. Only one mHealth solution has been implemented across an international network of hospitals [63,85], ASyMS, a phone-based, remote symptom monitoring system that was deployed and implemented in 13 cancer centers across 5 European countries (Austria, Greece, Ireland, Norway, and United Kingdom) [63].

#### Inner Setting

The inner setting refers to both structural characteristics that facilitate the implementation process and to dedicated activities activated by the recipient organizations along the way.

*Structural characteristics* of an organization, such as its age, size, and maturity, can significantly impact the effectiveness of mHealth interventions. Although information on these dimensions could not always be inferred from the selected papers, the type of clinical setting in which the study was being conducted was analyzed, although it did not seem discriminating.

*Implementation climate* is defined as the “absorptive capacity for change, shared receptivity of involved individuals to an intervention, and the extent to which use of that intervention will be rewarded, supported, and expected within their organization” [43]. In the context of mHealth apps, tensions for change resulting from perceived suboptimal situations can be observed. Patients with cancer went from being treated as in-patients to being increasingly and predominantly treated in outpatient settings. In this context, effective patient-clinician communication and facilitation with HCPs became key in the event of unforeseen symptoms and side effects, as when missing or not adequately provided, increased ED visits and hospitalization might follow [81,96]. The lack of HCPs supervision could be even more alarming in in-home administration regimens that require greater autonomy from the patients. Simultaneously, new therapeutic options are available. For instance, oral agents [9,48,52,56-58,65,76,80,82,​^90^,^91^,^94^,^111^,^122^] have become common today; however, their efficacy may be reduced owing to lack of adherence, erratic dosage intake, and inadequate self-management of adverse event self-management [91,122]. In addition, the growth in the uptake of mobile technologies also appeared to be connected to the need to reduce current health care spending [119]. Because of the economic implications of suboptimal medication adherence, such as increased risk of hospitalization and associated complication costs, app-based adherence interventions could mitigate this likelihood [48]. From the perspective of health care providers, mHealth could be seen as a way to make health systems more cost-effective [132]. Livingston et al [83] assessed the potential of an mHealth app in reducing the burden of screening and follow-up in busy clinics by freeing clinician time for those who need specialized follow-up [83]. According to Navarro-Alamán et al [86], managing patient symptomatology could require more than half the time spent by HCPs in monitoring the patient’s status. Shortages in health care resources were another factor that could foster the diffusion of mHealth solutions. Communicating with HCPs could be perceived as onerous [45], as pointed out in a study in which accessing well-trained pain therapists in-person appeared difficult and costly [77]. The imbalance between the number of clinicians available and the number of patients in need could be such that the latter are individually dedicated to only a few minutes of their clinician’s time [50]. All these factors suggest that health care models should evolve toward more convenient solutions for patients and more cost-effective solutions for the overall health system [49].

Adopting mHealth apps is perceived as a *relative priority* within organizations. Some studies showed that physicians were aware that their ability to evaluate patients’ symptoms was not optimal and acknowledged mHealth as a facilitator [64]. Not surprisingly, a survey of German health care providers showed high readiness to incorporate the use of mHealth apps into cancer treatment plans [120].

Regarding the *compatibility* of mHealth apps with the values of recipient organizations, openness from clinicians and patients to use mHealth as part of their routine could be observed [84]. Interoperability with existing IT systems and workflows was clearly preferred [87], and feasibility studies, including pilot testing, were typically used to demonstrate that an intervention could be integrated into clinical management. Interestingly, social factors, such as endorsements by trusted clinicians, likely influenced the perceived fit between an intervention and individuals [74].

*Organizational incentives and rewards* for using mHealth services were not systematically observed in the selected literature. Jacob et al [74] argued that app use could act as a tool to evaluate people and assign monetary rewards. A potential, yet indirect incentive was observed, which was an increased work-life balance resulting from fewer unscheduled consultations derived from correct app use [16].

#### Characteristics of Individuals

The likelihood of embracing a new health intervention also depends on the characteristics of the individuals who will use it. First, individuals’ *knowledge and beliefs about an intervention* can be good predictors of implementation effectiveness. In the context of mHealth, age was used as a proxy for individual recipients’ familiarity with and propensity to use digital health tools. In a large share of the selected papers (40/91, 44%), the observed mean age of the study participants ranged between 50 and 75 years. Nevertheless, as most participants routinely used smartphones [50,82,98,110,116], age did not seem to hamper their willingness to use mHealth services [16,50,58,73,89]. In addition, some studies have indicated that patients who are more inclined to use digital health solutions at large [62,73] or receive guidance [60] are more prone to use mHealth interventions. Patients’ attitudes toward digital technologies were also mentioned as an important factor in the acceptance of mHealth intervention [74,85,100].

The perception that individuals have about their ability to use a given intervention and how it changes over time falls under the *self-efficacy* and *individual stage of change* constructs [133]. Higher degrees of self-efficacy are associated with a greater willingness to embrace novel technologies [134]. Increasing self-efficacy is often among the primary goals of the selected studies [46,64,114,121,128,131]. Instruments such as the Stanford Inventory of Cancer Patient Adjustment scale were used to assess the self-efficacy of general health strategies during the cancer disease trajectory [114,131]. mHealth apps could support the patients better understand their symptoms and adverse events, thereby increasing their perceived safety and engagement with cancer therapy [16,49,73,80,94,95]. Severe side effects are a major concern for patients with cancer [47]. The willingness to cope better with cancer-related complications could increase the patient’s propensity to rely on mHealth interventions. Patients’ acceptability and usability were frequently assessed in the selected studies using study-specific or validated questionnaires (eg, Mobile Application Rating Scale questionnaire) [46,73,95,109], including scales that gauge the ease of use and perceived usefulness of a technology, such as the Technology Acceptance Model [8,72,84].

I*dentification with the organization* cannot be easily inferred from the selected papers. Pappot et al [88] reported that app users may not feel an added sense of belonging when using an app, thus potentially explaining the different benefits experienced by the treatment arm.

Finally, among *other personal attributes*, cultural views on smartphone use at work, such as the fear that colleagues might see it as a waste of time, were highlighted as potential barriers to mHealth use in the workplace by Jacob et al [74].

#### Process

Built on 4 dimensions (planning, engaging, executing, reflecting, and evaluating), *process* refers to the reliance on a well-defined implementation approach. This is the most difficult domain to define, measure, or evaluate in implementation research [135]. Appraisal of the implementation process was limited to a subset of study designs, excluding protocols or development studies. The study durations in RCTs and observational studies were limited (average 238 days; median 180 days; minimum 21 days; maximum 720 days). Although the design and development were extensively illustrated, rarely could the same level of detail be observed with respect to the implementation pathway. In the selected papers, no *opinion leaders*, *formally appointed implementation roles*, or *champions* are mentioned. Nurses seemed to be the stakeholders with the greatest potential to push mHealth uptake [66,73,100] and could be appointed as official reference persons for patients on any issues related to app use [12,52,55,59,61,66,67,77,87,94,106,111,129]. As for *external change agents*, recommendations from peer clinicians, medical societies, or social media channels could have an impact on the perception of mHealth [74], yet the appraisal of the long-term sustainability of the implementation process remains difficult, as these are general forces external to the organization [135]. Therefore, training was most frequently used to involve intended users, and participants were instructed on mHealth use by either the research team or dedicated clinical staff [9,12,16,46-48,50,51,53-55,57,59,67,69,73,80,91,95,111,119]. Dedicated meetings could allow for information exchange on implementation strategies, and easy access to technical support in case questions were deemed important in the process [100]. Technical information on the installation of the apps was sometimes provided as part of the studies [55,61,129], and integration in the hospital’s informative systems and workflows was also cited as an enabler to implementation [9,52,58,124].

Key barriers and enablers of mHealth uptake are illustrated in Table 3.

**Table 3:** Summary of key identified enablers and barriers to mobile health implementation.

CFIR^a^ construct and enablers		Barriers
**Intervention characteristics**		
	User-friendly interfaces Pretesting through small-scale pilot trials Patient’s and HCP’s^b^ involvement in the app development	Release of many subsequent app versions Data privacy
**Outer setting**		
	New patient needs (eg, need for constant monitoring, or real-time communication with HCPs) External policies and incentives fostering digital health Scarcity of resources and need to search more cos-effective ways to deliver health services	Unharmonized regulatory provisions across EU^c^ countries Tendency not to leverage on networks (ie, unrealized synergies of economies of scales
**Inner setting**		
	Interoperability with IT systems Workforce shortages New care pathways for cancer (eg, outpatient settings) Social endorsement (eg, peer referral)	HCPs’ perception of extra workload (eg, more data input) Clinician concern from following-up more patients Linkage between app uptake and incentives only possible at organizational level
**Characteristics of individuals**		
	Routine use of smartphones, regardless of age Positive attitude toward digital health	Cultural norms (eg, smartphone use in the workplace Perceived poorer communication with HCPs Weakened sense of identification with health service providers
**Process**		
	Training on app benefits and functioning Nurses’ active support	Unclear contribution of different stakeholders to implementation Implementation plans missing or poorly defined

^a^CFIR: Consolidated Framework for Implementation Research.

^b^HCP: health care professional.

^c^EU: European Union.

## Discussion

### Summary of Key Results

The overarching aim of this study was to investigate the determinants of mHealth uptake to inform the translation efforts of mHealth interventions in routine care. Studies illustrating the development, evaluation, and implementation of mHealth apps for cancer patients were considered, and information on barriers and enablers of app uptake was extracted following the CFIR scheme.

Many facilitators of app implementation in clinical settings have been identified. The involvement of patients and HCPs in app development has frequently been observed. Codevelopment was presented as a way to include desired mHealth features in early design efforts, to prevent unnecessary shortcomings, and activate a sense of ownership. These findings corroborated the idea that users should be intimately involved in the identification, design, and conduct phases of research, and not just be targets for the dissemination of study results [136]. An iterative development approach was often mentioned, as it ensured extensive usability testing during the development process.

As for implementation barriers, gradual rollouts and subsequent app version releases could be perceived as burdensome. From the provider’s perspective, mHealth could be referred to as a source of extra workload for the clinical staff. Conversely, factors characterizing providers, such as organizational leaders and management, staff, and culture, which can influence their ability to adapt and successfully use an intervention, were not systematically observed. From the user’s perspective, the fear of poorer patient-clinician interactions (eg, through remote monitoring) can diminish the sense of trust in the organization, in line with what was observed in prior works [137]. Although references to the outer setting (eg, laws and guidelines) were reported, mHealth was presented more as a way to address new or existing patient needs than as a way to respond to a given external pressure.

### Broader Implications

Although some of the findings discussed above are supported in previous research [138], and more broadly in the implementation science literature applied to DHTs, mHealth-specific dimensions resonated in this analysis. The peculiarities of mHealth, including the iterative nature of the corresponding interventions, frequent user interactions, a nonlinear relationship between technology use, engagement, and outcomes, implications at the organizational level, and challenges associated with genericization, distinguish apps from other DHTs [139]. For instance, compared with medical devices, typically evaluated through comparative evidence, studies on mHealth are often single-arm, noncomparative. Implementation hurdles related to system interoperability, data management, and patient privacy could appear to be more intricate for mHealth. Although these factors are reflective of the implementation challenges of DHTs, the distinctive features of mHealth seem to exacerbate their complexity.

mHealth will become increasingly important. On one hand smartphones are becoming increasingly prevalent and provide augmented functionalities (eg, cameras to capture high definition images of body parts). In contrast, demographic and epidemiological trends report a boom in chronic conditions, whose needs can be addressed by mHealth. Digitalization of the health care sector is a key priority in the political agenda, as confirmed by the expected massive capital injection in response to the COVID-19 pandemic. With more than €750 billion (US $798.38 billion), the next-generation European Union fund will invest a relevant share in promoting digital health, further boosting the development of mHealth apps. Although a stronger financial commitment is advocated [131], even in contexts where governments are directing huge health care spending to mHealth (eg, German DiGA), reimbursement policies do not always translate into actual clinicians’ prescriptions and are not a guarantee for users’ uptake [140]. Therefore, there is a need to adopt assessment frameworks for DHTs, including mHealth apps. Guidance on how to operationalize later implementation efforts is strongly advocated to avoid investing in technologies that are likely to be abandoned.

### Comparison With Prior Work

To our knowledge, this is the first review of the literature that uses a theory-guided framework to explore the determinants of mHealth implementation using a comprehensive approach in the area of cancer care. Other syntheses of primary studies mostly investigate the distinguishing features of mHealth [141,142] or their effectiveness in improving patient outcomes [143,144]. Studies illustrating the implementation initiatives in the area of mHealth are still limited and mainly document individual case studies. Although the field of implementation science has been growing, there is still a need to expand the use of implementation research to contribute to more effective public health and clinical practices [136]. Evidence suggests that theory-informed approaches to implementation science can enhance the translation and use of digital technologies in daily practice [145,146]. Under the lens of implementation science, Bardosh et al [138] conducted a qualitative evaluation of a single mHealth intervention addressing medication adherence and patient engagement. Heinsch et al [147] conducted a review of the theories that inform the implementation of eHealth interventions, and concluded that these are focused predominantly on predicting or explaining end user acceptance, and suggested that future research should test models that reflect the multidimensional, dynamic, and relational nature of the implementation process. Our work adds to the available literature by conducting a multidomain, multiple-stakeholder assessment of the determinants of mHealth implementation using the CFIR model. Rather than focusing more on a limited set of studies describing prevailing implementation research, our findings provide an integrated perspective on the factors that could influence the uptake and implementation of mHealth in clinical settings.

### Limitations

This study has several limitations. First, the papers selected for analysis were heterogeneous in terms of study characteristics (eg, purposes, study setting, design, duration, number, and types of participants). The decision to include a diverse range of studies was justified by the exploratory nature of scoping reviews [148] and stemmed from the observation that evidence from implementation research on mHealth solutions remains scarce. This was reflected in the search string, where implementation-related terms had to be. In addition, elaborating on implementation strategies, such as those described by the ERIC taxonomy Powell et al [149], seemed premature and was not performed. Although 29% (26/91) of the studies were RCTs, a proxy for evidence strength or quality, 24% (22/91) of the selected records had a sample size smaller than 20 patients, and 57% (52/91) were single-arm studies. Given this heterogeneity, a risk of bias assessment was not performed, although this is not unusual in scoping reviews [39,150]. Study heterogeneity also limits the possibility of performing meta-analyses on comparable outcomes. Finally, limiting the search to studies in English published since 2017 excluded a priori other potentially relevant earlier studies written in different languages.

### Conclusions

This review sheds light on the determinants of mHealth uptake in clinical practice, exploring the barriers and enablers of the implementation of cancer care apps using an established implementation science framework. It contributes to filling the knowledge gap by systematizing the dimensions that should be factored into when designing an implementation strategy for mHealth apps.

Future studies should investigate whether and how specific dimensions such as app development and deployment platforms could affect implementation-related elements. In addition, a core set of outcomes associated with successful implementation, measured in studies that discuss implementation initiatives including hybrid designs, should be developed [151]. Finally, future studies should complement the organizational perspective from the current work with a patient-oriented (user) view and investigate the relationship between patient-reported measures and implementation outcomes. In this regard, technology adoption models such as the Technology Acceptance Model [152] or the Unified Theory of Acceptance and Use of Technology [153] could be relevant theoretical starting points.

## Appendix

Multimedia Appendix 1 The 22-item Preferred Reporting Items for Systematic Reviews and Meta-Analyses extension for Scoping Reviews (PRISMA-ScR) checklist for scoping reviews.

Multimedia Appendix 2 Consolidated Framework for Implementation Research domains and constructs.

Multimedia Appendix 3 Overview of the descriptive statistics.

Multimedia Appendix 4 Characteristics of the selected studies.

## Abbreviations

CFIR: Consolidated Framework for Implementation Research
DHT: digital health technology
DiGA: Digital Health Applications
HCP: health care professional
mHealth: mobile health
PRISMA-ScR: Preferred Reporting Items for Systematic Reviews and Meta-Analyses extension for Scoping Reviews
RCT: randomized controlled trial
