# Supplementary material for: Evaluating Barriers and Facilitators to the Uptake of mHealth Apps in Cancer Care Using the Consolidated Framework for Implementation Research: Scoping Literature Review
Source: JMIR Cancer. 2023 Mar 30;9:e42092. doi: 10.2196/42092 (PMC10131717; doi:10.2196/42092)
Supplement: Multimedia Appendix 3 [file cancer_v9i1e42092_app3.docx]

*Overview of the descriptive statistics*

| **KEY CHARACTERISTICS OF INCLUDED RECORDS (n=89)** | | |
| --- | --- | --- |
| **RECORD CHARACTERISTICS** | **n** | **%** |
| **Record type** | | |
| Research article | 78 | 85.7 |
| Study protocol | 13 | 14.3 |
| **Country of study** | | |
| Europe | 43 | 47.3 |
| USA | 18 | 19.8 |
| China | 9 | 9.9 |
| South Korea | 6 | 6.6 |
| Rest of the world | 15 | 16.5 |
| **Publication year** | | |
| 2017 | 13 | 14.3 |
| 2018 | 13 | 14.3 |
| 2019 | 16 | 17.6 |
| 2020 | 26 | 28.6 |
| 2021  2022 | 19  4 | 20.9  4.4 |
| **Study design** | | |
| RCT | 26 | 28.2 |
| Mixed methods | 24 | 26.4 |
| Qualitative design | 12 | 13.2 |
| Pilot study | 11 | 12.1 |
| Other non-randomized clinical tirals | 7 | 7.7 |
| Pre-post | 3 | 3.3 |
| Quasi-experimental | 3 | 3.3 |
| Other | 5 | 5.5 |
| **Study arms** | | |
| Single-arm | 52 | 57.1 |
| Two-arm | 38 | 41.8 |
| Multiple-arm | 1 | 1.1 |
| **Sample Size** | | |
| 0 – 20 | 22 | 24.2 |
| 21 – 50 | 22 | 24.2 |
| 51 – 100 | 26 | 28.6 |
| 101 – 150 | 13 | 14.3 |
| More than 150 | 8 | 8.8 |
