# Supplementary material for: Evaluating Barriers and Facilitators to the Uptake of mHealth Apps in Cancer Care Using the Consolidated Framework for Implementation Research: Scoping Literature Review
Source: JMIR Cancer. 2023 Mar 30;9:e42092. doi: 10.2196/42092 (PMC10131717; doi:10.2196/42092)
Supplement: Multimedia Appendix 4 [file cancer_v9i1e42092_app4.docx]

*Characteristics of selected studies*

| **Reference (author, year)** | **Country** | **Record type** | **Study Design** | **Prospective/ Retrospective** | **Study arms** | **Study participants** | **App name** | **Cancer type** | **Treatment choice** | **Target users** |
| --- | --- | --- | --- | --- | --- | --- | --- | --- | --- | --- |
| Adam et al., 2020 | UK | Research article | Mixed-methods Study | Prospective | single-arm | n≤20 | Can-Pain | Bone/ Bone marrow | Strong opioids to alleviate pain | patients, clinicians, caregivers |
| Ahmed et al., 2021 | Canada | Research article | Mixed-Methods Study | Prospective | single-arm | 20<n≤50 | BELONG | Gynaecologic | N/A | patients, clinicians, caregivers |
| Alboughobeish et al., 2017 | Iran | Research article | Open-label clinical trial | Prospective | two-arm | 20<n≤50 | N/A | General | Chemotherapy | patients |
| Ali et al., 2019 | Singapore | Research article | Qualitative Design | Prospective | single-arm | n≤20 | MedFC | General | Oral Treatment | patients, clinicians |
| Armstrong et al., 2017 | Canada | Research article | RCT | Prospective | two-arm | 50<n≤100 | QoC Health Inc App | Breast | Ambulatory breast reconstruction | patients, clinicians |
| Bae et al., 2018 | South Korea | Research article | Pilot Study | Prospective | single-arm | 100<n≤150 | PRO-SMART | General | Chemotherapy | patients, clinicians |
| Benze et al., 2019 | Germany/ Switzerland | Research article | Any other clinical trial | Prospective | single-arm | 20<n≤50 | MeQoL | Solid tumours | N/A | patients, clinicians |
| Berry et al., 2018 | USA | Research article | Pilot Study | Prospective | single-arm | 50<n≤100 | iCancerHealth | Gastric and Colon | Oral Treatment | patients, clinicians |
| Biran et al., 2020 | USA | Research article | Mixed-Methods Study | Prospective | single-arm | n≤20 | Medocity Home Health | Gastric and Colon | N/A | patients, clinicians |
| Birkhoff et al., 2018 | USA | Research article | Mixed-Methods Study | Prospective | single-arm | 20<n≤50 | Health Storylines app | General | Radiotherapy | patients, clinicians |
| Breen et al., 2016 | Australia | Research article | Mixed-methods Study | Prospective | single-arm | n≤20 | ASyMS-H | Hematology | Chemotherapy | patients, clinicians |
| Ciani et al., 2018 | Italy | Study Protocol | RCT | Prospective | two-arm | 100<n≤150 | LuCApp | Lung | Chemotherapy | patients, clinicians |
| Collado-Borrell et al., 2020a | Spain | Research article | Qualitative Design | Prospective | single-arm | 50<n≤100 | e-OncoSalud | Hematology | Oral antineoplastic agents | patients, clinicians, pharmacists |
| Collado-Borrell et al., 2020b | Spain | Research article | Cross-sectional analysis | Retrospective | single-arm | 20<n≤50 | e-OncoSalud | Hematology | Oral antineoplastic agents | patients, clinicians, pharmacists |
| Collado-Borrell et al., 2020c | Spain | Research article | Quasi-Experimental Study | Mixed | two-arm | 100<n≤150 | e-OncoSalud | General | Oral antineoplastic agents | patients, clinicians, pharmacists |
| Crafoord et al., 2020 | Sweden | Research article | Mixed-Methods Study | Prospective | two-arm | 100<n≤150 | Interaktor | General | Chemotherapy | patients, clinicians |
| Crawford et al., 2019 | USA | Research article | Mixed-methods Study | Prospective | single-arm | n≤20 | mPACT | General | Oral Treatment | patients, clinicians |
| Daly et al., 2020 | USA | Research article | Pilot Study | Prospective | single-arm | 50<n≤100 | InSight Care | Solid tumour or lymphoma | Antineoplastic | patients, clinicians |
| Di R. et al., 2018 | China | Research article | Non-randomized controlled study | Prospective | two-arm | 100<n≤150 | N/A | Nasopharyngeal Carcinoma | Concurrent radiotherapy and chemotherapy | patients |
| Diehl et al., 2022 | USA | Research article | Pre-post Study | Mixed | single-arm | 20<n≤50 | MobiMD | Other | Abdominal surgery | patients, clinicians |
| El Shafie et al., 2018 | Germany | Study Protocol | Pilot Study | Prospective | single-arm | 20<n≤50 | OPTIMISE-1 survey app | Thoracic or Pelvic | Radiotherapy | patients, clinicians |
| Ellen et al., 2021 | UK | Research article | Mixed-methods Study | Prospective | single-arm | 50<n≤100 | OurBrainBank | Brain | N/A | patients, caregivers |
| Elsbernd et al., 2018 | Denmark | Study Protocol | Mixed-Methods Study | Prospective | single-arm | 50<n≤100 | Kræftværket | Hematology | N/A | patients |
| Fishbein et al., 2017 | USA | Study Protocol | Mixed-Methods Study | Prospective | single-arm | n≤20 | CORA | General | Oral Treatment | patients, clinicians |
| Furlong et al., 2019 | Ireland | Research article | Mixed-Methods Study | Prospective | single-arm | 50<n≤100 | ASyMS | Gastric and Colon | Chemotherapy | patients, clinicians |
| Giannoula et al., 2020 | Greece | Study Protocol | Pilot Study | Prospective | two-arm | 20<n≤50 | DTC app | Thyroid | Surgery and thyroid stimulating hormone (TSH)-suppressive doses | patients, clinicians |
| Grašič Kuhar et al., 2020 | Slovenia | Research article | Non-randomized controlled study | Prospective | two-arm | 50<n≤100 | mPRO Mamma | Breast | Chemotherapy | patients |
| Greer et al., 2020 | USA | Research article | RCT | Prospective | two-arm | n>150 | N/A | General | Oral Treatment | patients |
| Gustavell et al., 2019 | Sweden | Research article | Qualitative Design | Prospective | single-arm | n≤20 | Interaktor | General | Pancreatico-duodenectomy | patients, clinicians |
| Gustavell et al., 2019 | Sweden | Research article | Mixed-Methods Study | Prospective | two-arm | 50<n≤100 | Interaktor | Pancreatic | Pancreatico-duodenectomy | patients |
| Gustavell et al., 2020 | Sweden | Research article | Descriptive Study | Prospective | single-arm | 20<n≤50 | Interaktor | Pancreatic | Pancreatico-duodenectomy | patients, clinicians |
| Handa et al., 2020 | Japan | Research article | RCT | Prospective | two-arm | 50<n≤100 | BPSS | Breast | Chemotherapy | patients |
| Hanghøj et al., 2020 | Denmark | Research article | Qualitative Design | Prospective | single-arm | n≤20 | Kræftværket | General | N/A | patients |
| Hou et al., 2020 | Taiwan | Research article | RCT | Prospective | two-arm | 100<n≤150 | BCSMS | Breast | N/A | patients |
| Hyatt et al., 2020 | Australia | Research article | Mixed-Methods Study | Prospective | single-arm | 20<n≤50 | SecondEars | General | N/A | patients |
| Jacob et al., 2019 | UK | Research article | Qualitative Design | Prospective | single-arm | n≤20 | ONCOassist | General | N/A | clinicians |
| Ji et al., 2019 | South Korea | Research article | Other types of clinical trials | Prospective | two-arm | 50<n≤100 | efil breath | Lung | Pulmonary rehabilitation; radio- and chemotherapy | patients, clinicians |
| Karaaslan-Eser et al., 2021 | Turkey | Research article | RCT | Prospective | two-arm | 50<n≤100 | OKTED | General | Oral Treatment | patients |
| Karsten et al., 2021 | Germany | Study Protocol | RCT | Prospective | two-arm | n>150 | PatientConcept | Breast | N/A | patients, clinicians |
| Kelleher et al., 2021 | USA | Research article | RCT | Prospective | two-arm | n>150 | mPSCT (Mobile Pain Coping Skills Training) | Breast | N/A | patients, clinicians |
| Kim et al., 2018 | South Korea | Research article | RCT | Prospective | two-arm | 50<n≤100 | ILOVEBREAST | Breast | Chemotherapy | patients |
| Kneuertz et al., 2020 | USA | Research article | Pilot Study | Prospective | single-arm | 20<n≤50 | SeamlessMD | Lung | Surgery | patients |
| Kongshaug et al., 2021 | Norway | Research article | Qualitative Design | Prospective | single-arm | n≤20 | N/A | Breast | Oral Treatment | patients |
| Langius Elköf et al., 2017a | Sweden | Research article | Qualitative Design | Prospective | single-arm | 50<n≤100 | Interaktor | Gastric and Colon | Radiotherapy | patients |
| Langius-Eklöf et al., 2017b | Sweden | Study Protocol | RCT | Prospective | two-arm | 100<n≤150 | Interaktor | General | Chemotherapy | patients, clinicians |
| Lidington et al., 2020 | UK | Study Protocol | RCT | Prospective | two-arm | 100<n≤150 | Owise | Breast | N/A | patients |
| Lin et al., 2021 | China | Study Protocol | RCT | Prospective | two-arm | n>150 | Mobile Gynaecological Cancer Support (MGCS) | Head and neck | Chemotherapy | patients, clinicians |
| Linder et al., 2019 | USA | Research article | Other | Prospective | single-arm | 20<n≤50 | Dosecast Pro | General | Oral Treatment | patients |
| Livingston et al., 2019 | Australia | Research article | RCT | Prospective | two-arm | 50<n≤100 | ACE | General | N/A | patients |
| Maguire et al., 2020 | UK | Research article | Mixed-Methods Study | Prospective | single-arm | n≤20 | ASyMSmeso | Thyroid | N/A | patients, clinicians |
| Moradian et al., 2018 | Canada | Research article | Mixed-Methods Study | Prospective | single-arm | n≤20 | ASyMS | General | Chemotherapy | patients, clinicians |
| Navarro-Alaman et al., 2020 | Spain | Research article | Mixed-Methods Study | Prospective | single-arm | 20<n≤50 | Close2U | General | N/A | patients, clinicians |
| Ngo et al., 2020 | USA | Research article | RCT | Prospective | two-arm | 50<n≤100 | Personal Health Network (PHN) | General | Chemotherapy | patients |
| Nyman et al., 2017 | Sweden | Research article | Qualitative Design | Prospective | single-arm | 20<n≤50 | Interaktor | General | Radiotherapy | patients, clinicians |
| Pappot et al., 2019 | Denmark | Research article | Pre-post Study | Prospective | two-arm | n≤20 | Kræftværket | General | N/A | patients |
| Park et al., 2019 | South Korea | Research article | Pilot Study | Prospective | single-arm | 50<n≤100 | efil breath | Lung | Pulmonary rehabilitation; radio- and chemotherapy | patients, clinicians |
| Passardi et al., 2017 | Italy | Research article | Mixed-Methods Study | Prospective | single-arm | 50<n≤100 | Onco-TreC | General | Oral Treatment | patients, clinicians |
| Passardi et al., 2022 | Italy | Research article | Prospective Training-Validation Trial | Prospective | single-arm | 20<n≤50 | ONCO-TreC | General | Oral Treatment | patients, caregivers, clinicians |
| Peltola et. al 2021 | Finland | Research article | Pilot Study | Prospective | single-arm | 50<n≤100 | Noona | Gynaecological | Radiotherapy | patients, clinicians |
| Peng et al., 2020 | China | Research article | RCT | Prospective | two-arm | n>150 | WeChat | General | N/A | patients, clinicians |
| Pereira-Salgado et al., 2017 | Australia | Research article | Mixed-Methods Study | Prospective | single-arm | n≤20 | REMIND | Hematology | Oral tyrosine kinase inhibitor therapy | patients, clinicians |
| Petrocchi et al | Switzerland | Research article | Mixed-Methods Study | Prospective | single-arm | n≤20 | CSSI | Breast | N/A | patients |
| Ponder et al., 2021 | USA | Research article | Mixed-Methods Design | Prospective | single-arm | 20<n≤50 | Manage My Surgery (MMS) | Breast | Hematopoietic stem cell transplantation (HCT) | patients, clinicians |
| Racioppi et al., 2020 | USA | Research article | Pilot Study | Prospective | single-arm | 20<n≤50 | Bone Marrow Transplantation (TRU-BMT) app | Breast | Transplantation (e.g. stem cell) | patients |
| Richards et al., 2021 | UK | Research article | Qualitative Design | Prospective | single-arm | 20<n≤50 | N/A | Breast | N/A | patients, clinicians |
| Salmani et al., 2021 | Iran | Research article | Qualitative Design | Prospective | single-arm | n≤20 | ColorectAlong | Gastric and Colon | N/A | patients |
| Sauer et al., 2020 | Germany | Study Protocol | RCT | Prospective | two-arm | 50<n≤100 | SOFIA App | General | Immune checkpoint therapy (ICT) | patients, clinicians |
| Seljelid et al., 2021 | Norway | Research article | Qualitative Design | Prospective | single-arm | n≤20 | InvolveMe | Thyroid | Transplantation (e.g., stem cell) | patients, clinicians |
| Soh et al., 2018 | South Korea | Research article | Mixed-Methods Study | Prospective | single-arm | n>150 | Life Manager | Gastric and Colon | Chemotherapy | patients, clinicians |
| Soh et al., 2019 | South Korea | Research article | RCT | Prospective | two-arm | 20<n≤50 | Go-breath | Gastric and Colon | Other | patients |
| Somers et al., 2018 | USA | Research article | RCT | Prospective | two-arm | 20<n≤50 | mPSCT (Mobile Pain Coping Skills Training) | Breast | Hematopoietic Stem Cell Transplantation | patients |
| Sprave et al., 2020 | Germany | Study Protocol | RCT | Prospective | two-arm | 50<n≤100 | N/A | Breast | Chemotherapy | patients, clinicians |
| Sun et al., 2017 | China | Research article | RCT | Prospective | two-arm | 20<n≤50 | Intelligent Pain Management System (IPMS) | General | N/A | patients, clinicians |
| Sun et al., 2021 | UK | Research article | Mixed-Methods Study | Prospective | single-arm | n≤20 | N/A | Oesophageal | N/A | patients, clinicians |
| Sundberg et al., 2017 | Sweden | Research article | Non-randomized controlled study | Prospective | two-arm | 100<n≤150 | Interaktor | General | Radiotherapy | patients, clinicians |
| Sundberg et al., 2020 | Sweden | Research article | Quasi-Experimental Study | Mixed | two-arm | 100<n≤150 | Interaktor | General | Radiotherapy | patients |
| Trojan et al., 2021 | Switzerland | Research article | Mixed-Methods Study | Mixed | two-arm | n>150 | Consilium Care | General | N/A | patients, clinicians |
| Walle et al., 2020 | Germany | Research article | RCT | Prospective | two-arm | 50<n≤100 | Minxli—Arzt via Video Chat | Solid tumors | Systemic cancer therapy | patients, clinicians |
| Wan et al, 2021 | Singapore | Research article | Qualitative Design | Prospective | single-arm | n≤20 | iCanManage | General | Surgery | patients, caregivers |
| Wang et al., 2020 | Taiwan | Research article | Quasi-Experimental Study | Prospective | two-arm | 50<n≤100 | N/A | Other | Surgery | patients |
| Wright et al., 2018 | USA | Research article | Pilot Study | Prospective | single-arm | n≤20 | Beiwe | Other | Chemotherapy | patients, clinicians |
| Wu et al., 2018 | USA | Research article | Pre-post Study | Prospective | single-arm | 20<n≤50 | Dosecast | General | Oral Treatment | patients |
| Yanez et al., 2019 | USA | Study Protocol | RCT | Prospective | two-arm | 50<n≤100 | My Guide for Breast Cancer Treatment | Breast | Surgery and adjuvant therapy | patients |
| Yang et al., 2019 | China | Research article | RCT | Prospective | two-arm | 50<n≤100 | Pain Guard | General | N/A | patients, clinicians |
| Yu et al., 2021 | China | Research article | Cohort Study | Retrospective | more arms | n>150 | full-course management system | General | Multidisciplinary treatment, including chemotherapy, radiotherapy, endocrine and targeted therapy | patients, clinicians |
| Zhu et al., 2017 | China | Research article | RCT | Prospective | two-arm | 100<n≤150 | Breast-Cancer-Support (BCS) | Breast | Chemotherapy | patients, clinicians |
| Zhu et al., 2017 | China | Study Protocol | RCT | Prospective | two-arm | 100<n≤150 | Breast-Cancer-Support (BCS) | Breast | Chemotherapy | patients, clinicians |
| Zhu et al., 2020 | China | Research article | RCT (secondary data analysis) | Retrospective | single-arm | 50<n≤100 | Breast-Cancer-Support (BCS) | Breast | Chemotherapy | patients, clinicians |
| Zini et al., 2019 | Italy | Research article | Pilot Study | Prospective | single-arm | n≤20 | HeNeA | General | Chemotherapy | patients, clinicians |
| Hochstenbach et al., 2017 | Netherlands | Research article | Mixed-Methods Study | Prospective | single-arm | n≤20 | N/A | General | N/A | patients, nurses |
| Kondylakis et al., 2020 | German/ Italy | Research article | Mixed-Methods Study | Prospective | single-arm | 100<n≤150 | iManageCancer | Breast/  Prostate | N/A | patients, clinicians |
